# Advances in iron deficiency and iron-related arrhythmias and cardiovascular diseases

**DOI:** 10.3389/fcvm.2025.1573095

**Published:** 2025-07-11

**Authors:** Sung Il Im

**Affiliations:** Division of Cardiology, Department of Internal Medicine, Kosin University College of Medicine, Busan, Republic of Korea

**Keywords:** iron deficiency, atrial fibrillation, cardiovascular disease, anemia, arrhythima

## Abstract

Iron deficiency (ID) is common in patients with cardiovascular disease. Up to 60% of patients with coronary artery disease and even higher percentages of patients with heart failure (HF) or pulmonary hypertension have ID. However, the evidence for an association between ID including anemia and arrhythmias, particularly atrial fibrillation (AF) is less clear. The prevalence of ID increases with the severity of cardiac and renal dysfunction and would be more common in women. Increased blood loss due to antithrombotic therapy or gastrointestinal or renal disease and insufficient dietary iron intake, reduced iron absorption secondary to low-grade inflammation associated with congestion or reduced gastric acidity may cause ID. Both anemia and ID are associated with poor clinical outcomes, each may confer risk factors independently. There is growing evidence that ID is an important therapeutic target in patients with HF with reduced ejection fraction (HFrEF), even in the absence of anemia. Intravenous ferric carboxymaltose improved symptoms, ID-related quality of life, and exercise capacity and reduced hospitalizations for worsening HF in patients with HFrEF even mildly reduced EF (<50%). ID is easy to treat and effective in patients with HFrEF. These patients should be investigated for possible ID. Malnutrition has also been linked to cardiovascular disease. Both selenium and iron deficiencies have been associated with worse clinical outcomes in patients with HF. And selenium deficiency was associated with new-onset AF in nonsmoking participants. Interventional studies investigating the effects of optimizing the micronutrient status in at-risk populations are needed to assess causality, especially in those with ID. These recommendations may be extended to those populations based on evidence from future clinical trials.

## 1 Introduction

Iron is required by all organ systems for a variety of metabolic processes, such as erythropoiesis, mitochondrial function, oxygen transport, cardiac and skeletal muscle metabolism, immune and nervous systems, inflammatory response, and lipid metabolism (1).

Iron deficiency (ID) is common (2), and recent trials have shown it to be an important treatment target in patients with heart failure (HF) with reduced ejection fraction (HFrEF) (3, 4). However, ID appears to be common in a wide range of cardiovascular (CV) diseases. This review aimed to summarize the available data from epidemiological, and clinical studies on ID of arrhythmias and cardiovascular diseases. We followed the PRISM A guidelines and registration information (5).

## 2 Definition of iron deficiency

Anemia is a hematological disorder caused by a deficiency of red blood cells, most commonly defined as a decrease in hemoglobin concentration (6). Estimates of anemia prevalence vary by gender and age. Globally, the prevalence of anemia is estimated to be 10%–30% in non-pregnant women, 11%–12% in men, and 20%–24% in the elderly (7, 8). Among the various causes, anemia due to ID is the most common. Likewise, the prevalence of ID varies by race, gender, and age. In developing countries, the prevalence of ID can be as high as 41%–63% of women and 13% of men, whereas ID anemia can occur in 20%–39% of women and 4% of men (9). However, because comprehensive data are limited, these figures may underestimate the true burden of ID, especially non-anemic ID. There are numerous definitions and guidelines for diagnosing ID (10). Iron stores can be obtained from serum iron, ferritin, and transferrin levels (11). However, due to the nature of ferritin as an acute phase reactant, elevated serum ferritin levels also occur in other chronic inflammatory diseases, regardless of iron status. In chronic inflammatory conditions, serum ferritin production increases 2.5-fold (12). In these cases, additional evidence of low transferrin saturation under 20% and assessment of inflammatory markers (e.g., erythrocyte sedimentation rate and C-reactive protein) are needed to better assess ID (12, 13). In particular, a ferritin level of less than 100 ug/L or a combination of a ferritin level between 100 and 299 ug/L and a transferrin saturation of less than 20% are now guidelines recommended criteria for the diagnosis of ID in patients with heart failure. As explained below.

## 3 What mechanisms of chronic inflammation make iron deficiency?

Iron supplementation is thought to be beneficial in part because HF, similar to chronic kidney disease, cancer, and other inflammatory diseases, is associated with increased systemic inflammation and subsequent abnormal homeostasis of systemic iron (14, 15). Our understanding of the potential mechanisms of this phenomenon, termed functional ID, has evolved from several observations in chronic inflammatory diseases. Pro-inflammatory cytokines, such as interleukin-6, mediate the production and release of hepcidin (16). Hepcidin is a peptide that regulates the storage and release of iron by mediating the activity of iron proteins and iron transport proteins (4). In chronic inflammatory diseases, overexpression of hepcidin contributes to ID primarily by reducing iron protein levels and trapping iron in duodenal enterocytes and macrophages. Additionally, interferon-*γ*, lipopolysaccharide, and tumor necrosis factor-α upregulate the expression of divalent metal transporter 1, thereby increasing iron uptake in macrophages (17). Thus, abnormally high uptake and retention of iron occurs within the storage cells of the reticuloendothelial system, which limits iron availability for erythroid progenitor cells and erythropoiesis. Finally, inflammatory cytokines such as interleukin-1 and tumor necrosis factor-α also contribute to final anemia by suppressing erythropoietin expression and increasing erythrocyte phagocytosis (18). Therefore, chronic inflammatory diseases such as HF reduce the amount of iron available for erythropoiesis, resulting in a multifaceted pathophysiological process that precedes the development of anemia. This reduction in iron availability supports the benefit of intravenous iron supplementation in patients with heart failure (19). Notably, oral iron supplementation has not been shown to be beneficial, unlike intravenous replacement. Oral administration of iron is generally inadequate to prevent sequestration of stored iron in enterocytes and macrophages due to poor compliance and limited gastrointestinal absorption through mucosal edema (20).

## 4 What evidences of heart failure are related with iron deficiency and anemia?

**Figure 1:**
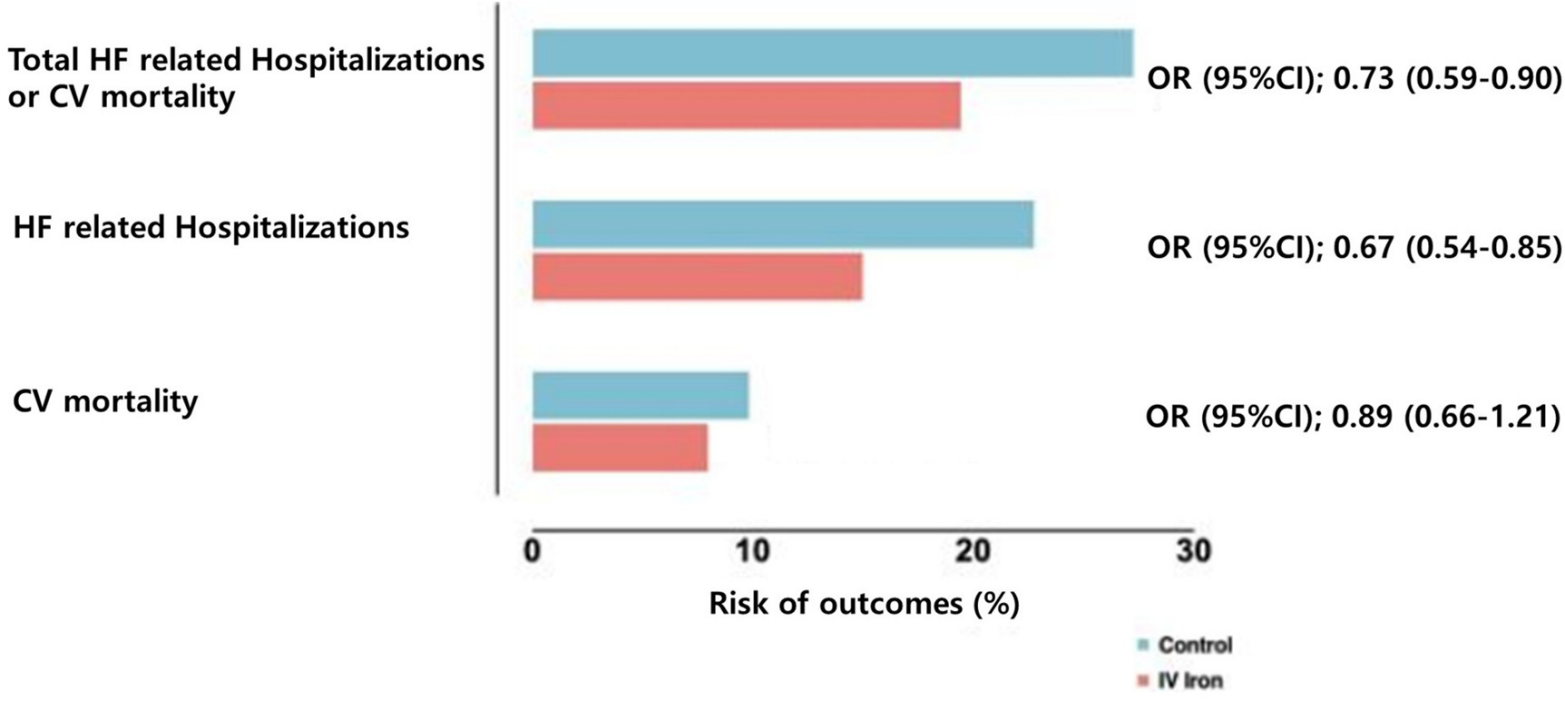
Risk of cardiovascular outcomes with intravenous iron vs. placebo. Data from Graham et al. (45) pooling data from multiple randomized controlled trials on intravenous iron supplementation in HF with iron deficiency. HF, heart failure; CV, cardiovascular; OR, odds ratio; CI, confidence interval.

Iron supplementation is an established treatment for heart failure patients with comorbidities (Figure 1). Up to half of patients with HFrEF have evidence of ID (21), and the prevalence may be even higher in HF patients with preserved ejection fraction up to 60% (22). Furthermore, this high prevalence of ID occurs even in the absence of anemia (23). Although treatment of anemia and ID with subcutaneous erythropoietin and oral iron supplementation has not been shown to be beneficial, trials of intravenous iron supplementation have shown a consistent benefit. In FAIR-HF trial, which recruited HF patients with ID, showed that intravenous ferric carboxymaltose had a significant benefit compared to placebo for improvements in patient global assessment, the 6-min walk test, and quality of life (4). Importantly, these benefits were similar, regardless of whether the patients were anemic or not. The previous study also demonstrated that ferric carboxymaltose resulted in improvements in the 6-min walk test, New York Heart Association class, patient global assessment, and health-related quality of life compared to placebo (CONFIRM-HF trial) (24). And, the intravenous iron supplementation treatment group maintained and decreased maximal oxygen consumption compared to the control group at 24-week follow-up (EFFECT-HF trial) (23). Moreover, intravenous iron supplementation reduces recurrent hospitalization after discharge for acute HF (AFFIRM-AHF trial) (25). These are summarized about previous randomized controlled trials on iron deficiency in heart failure in Table 1.

**Table 1:** Summary of previous randomized controlled trials on iron deficiency in heart failure.

Valuables	FAIR-HF	CONFIRM-HF	EFFECT-HF	AFFIRM-AHF
Patients (*n*)	459	301	172	1,108
Duration (weeks)	24	52	24	52
Gender (male, %)	47	53	75	55
Age, median (years)	67	69	63	71
Iron treatment vs. control	IV FCM vs. Placebo	IV FCM vs. Placebo	IV FCM vs. Placebo	IV FCM vs. Placebo
Inclusion criteria				
NYHA class	II–III	II–III	II–III	I–IV
LVEF (%)	<45%	<45%	<45%	<50%
ID definition	Current ESC HF Guidelines^a^	Current ESC HF Guidelines^a^	Current ESC HF Guidelines^a^	Current ESC HF Guidelines^a^
Hb (g/dl)	9.5–13.5	<15	<15	<8–15
Primary outcomes	PGA at follow-up NYHA at follow-up	Δ 6MWD	Δ peak VO2	Recurrent HFHosp or CV death
Key secondary outcomes	ΔPGA Δ6MWD ΔEQ-5D ΔKCCQ	ΔNYHA ΔPGA ΔFatigue score ΔKCCQ ΔEQ-5D	ΔVE/VCO2 ΔNYHA ΔPGA ΔNT-proBNP	Recurrent CV Hosp or CV death CV death Total HF hosp Time-to-first HF hosp or CV death

Outcomes significantly improved by iron supplementation are reported.

6MWD, 6 min walking distance; FCM, ferric carboxymaltose; IV, intravenous; LVEF, left ventricular ejection fraction; ID, iron deficiency; Hb, haemoglobin; HF, heart failure; PGA, patient global assessment; NYHA, New York Heart Association; KCCQ, Kansas City Cardiomyopathy Questionnaire; NT-proBNP, N-terminal pro-B-type natriuretic peptide; VE/VCO2, ventilation/carbon dioxide production; TSAT, transferrin saturation; EQ-5D, European Quality of Life Five Dimension; CV, cardiovascular; hosp, hospitalization; ESC, European Society of Cardiology.

^a^ Ferritin < 100 ng/ml OR ferritin 100–299 ng/ml AND TSAT <20%.

## 5 What is the potential role of anemia and iron deficiency for arrhythmia including atrial fibrillation?

The role of anemia and ID in patients with heart failure is well known. However, relatively little research has been done on the impact this condition may have on patients with atrial fibrillation (AF). However, the high prevalence of AF once again underlines the susceptibility of this population to arrhythmias. This occurs despite the fact that AF and HF commonly coexist, share major risk factors, have similar pathophysiological mechanisms, and predispose to each other. Both conditions have similar consequences, including reduced cardiac output, oxygen uptake, and maximal work capacity (26). Moreover, emerging evidence suggests that inflammation plays an important role in the pathogenesis of AF. For example, studies have shown that patients with AF have increased lymphocyte cell activity, increased myocyte death, increased inflammatory markers, and an increased neutrophil/lymphocyte ratio compared to control patients without AF (27). Signaling pathways present in inflammatory diseases can also induce atrial remodeling and limit atrial conduction through the mediation of matrix metalloproteinase (MMP) 2 and MMP-9 (28). Finally, anemia and ID can lead to severe myocardial hypertrophy and ventricular dilatation, which may predispose patients to HF and AF (29). Therefore, the interrelationship between AF, HF, and inflammation raises the strong possibility that the association between anemia and ID in AF may be similar to that observed in HF.

To date, there is limited research assessing the prevalence of anemia in patients with AF (30–32). In subgroup analysis of AFCAS registry, 30% of patients with atrial fibrillation with measurable hemoglobin who underwent coronary stenting were anemic (33). This prevalence was similar to the prevalence in another Danish registry study that reported a prevalence of 34% in a population of all AF patients (34). In contrast, the prevalence was 12% in another study, suggesting some variation among different AF populations (35). A recent meta-analysis of available data from 28 studies found that the weighted proportion of AF patients with anemia was 16% (36).

Previous study (Danish registry) also found that anemic patients had a significantly increased risk of major bleeding events, stroke, thromboembolic events, and all-cause mortality compared with non-anemic patients with AF (34). Two analyzes of individuals from controlled trials of various oral anticoagulants also found an association with anemia and increased risk of major bleeding and all-cause death (35, 37). In previous meta-analysis, anemia was associated with a 78% increased risk of all-cause mortality, and every 1 g/dl decrease in hemoglobin was associated with a 24% increased risk (36). Furthermore, anemia was also associated with a 15% increased risk of stroke or systemic thromboembolism and a 78% increased risk of major bleeding. Several recent cohort studies have reported an association between anemia and risk of hospitalization for heart failure (30, 38). And, at least one study suggested that anemia may be associated with clinical recurrences of AF (39). In this study, patients with anemia had a higher incidence of post-ablation AF than patients without anemia. This association requires replication but is likely due to an adverse effect of anemia on atrial remodeling.

Nonetheless, these studies on anemia in patients with AF raise the potential relevance of anemia in subsequent complications. However, studies assessing the effects of anemia on other AF symptoms such as functional capacity and exercise tolerance are limited. Recent study reported the quality of life using a validated questionnaire among AF patients with/without anemia. Although quality of life at baseline did not appear to be different, patients with anemia did not improve their quality of life as significantly as patients without anemia at follow-up (30).

**Figure 2:**
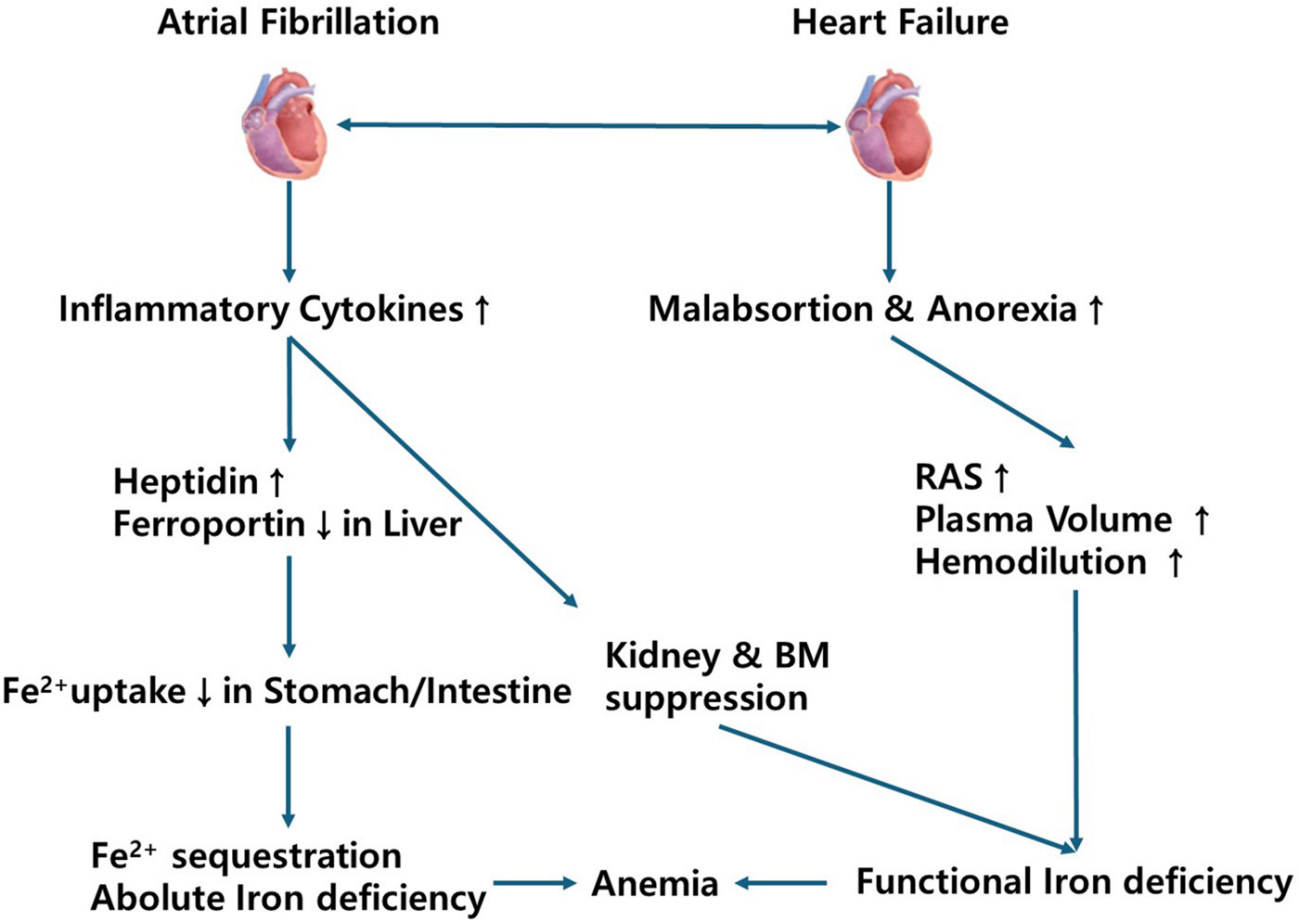
Potential mechanisms linking atrial fibrillation, heart failure, iron deficiency, and anemia. RAS, renin angiotensin system; BM, bone marrow.

These associations were biologically plausible. The impact of anemia on mortality may be mediated by the established association between anemia and other adverse events such as stroke, HF, and hemorrhage (40, 41). Conversely, anemia may be an indicator of general frailty, the latter potentially causing confounding. However, given the magnitude of the reported association, further investigation would be needed into the prognostic impact of anemia in AF and whether treatment of this comorbidity is beneficial. Potential mechanisms linking atrial fibrillation, heart failure, iron deficiency, and anemia are shown Figure 2.

## 6 What is clinical link between iron deficiency and atrial fibrillation?

There is a paucity of data regarding ID in patients with AF. Previous retrospective study has been published that analyzed data on ID in patients with AF (42). In this study, using a diagnostic threshold of ferritin level less than 100 ug/L, or a combination of a ferritin level between 100 and 299 ug/L and transferrin saturation under 20%, 47.6% of individuals with AF had ID. And, the prevalence of ID appeared to be higher in patients with permanent AF than in those with paroxysmal or persistent AF (43). A recent large-scale analysis of a national sample of inpatients found that 2.5% of patients hospitalized with atrial fibrillation (AF) were diagnosed with ID anemia (31). In cross-sectional analyses, ID anemia was associated with a longer length of stay and worse inpatient outcomes (e.g., myocardial infarction, renal injury, and vasopressor/ventilation requirement) excluding mortality. Despite its significant size, the prevalence reported in this study is likely underestimated due to the use of hospital coding data. As is the case with heart failure and other cardiovascular diseases, additional studies in diverse populations are needed to better characterize the prevalence of ID and ID anemia and to evaluate the potential relevance of these conditions to atrial fibrillation symptoms and complications.

## 7 Clinical significance and future treatment directions of the relationship between iron deficiency and atrial fibrillation

There is evidence that patients with AF may have significant rates of anemia and intellectual disability. Moreover, anemia appears to be associated with poor clinical outcomes in patients with AF. However, data on ID in AF are limited, preliminary findings suggest that the prevalence of ID may be non-significant or may also be correlated with severity and clinical outcome of AF. Although there is clearly a paucity of reports in this area, and these limited data should be interpreted with caution at this point in time. These initial findings suggest a strong possibility that anemia and ID may represent therapeutic targets for patients with AF. Moreover, this potential is supported by the close interrelationship between AF and HF, for which the role and benefits of iron supplementation have been established.

The RESAFE-HF trial is designed to further clinically evaluate the effect treating ID with intravenous ferric carboxymaltose (FCM) has on the arrhythmic burden of patients with HFrEF and Cardiac implantable electronic devices (CIEDs), while confirming via real-world data the benefits as for heart contractility, functional status, quality of life, HF hospitalizations, and survival (44). Several design decisions render RESAFE-HF uniquely suited to explore the effect of IV FCM on the arrhythmic burden of patients with HFrEF and ID. Importantly, only patients with implanted CIEDs were included in the study, which is expected to aid in the investigation of the effect of IV FCM in the arrhythmic burden of HFrEF patients in the aspect of that most CIEDs today hold a record of all atrial and ventricular tachycardia that fulfil sensor criteria. Thus, a reliable estimation of patients' arrhythmic burden can be derived by interrogating these devices. Eventhough, there is little data to support for those aforementioned theories. Therefore, future observational studies should provide additional estimates of the prevalence of anemia and ID in patients with AF, clarify the association of anemia and ID with clinical outcomes, and characterize the impact of anemia and ID on AF symptoms and functional capacity. Importantly, these studies were conducted in patients with and without heart failure, which provides insight into the potentially confounding effects of this condition. If this line of investigation proves promising, clinical trials to modify ID may be worthwhile. If successful, this may lead to routine screening and treatment of anemia and ID in patients with AF, as evaluated in the context of HF. Considering the increasing burden of AF worldwide, anemia and ID may be novel treatment strategies to evaluate in future studies. These are summarized about future directions of research and treatments for iron deficiency and arrhythmia in Table 2.

**Table 2:** Future directions of research and treatments for iron deficiency and arrhythmia.

Valuable about	Directions & goals
Prevalence & Incidence	Additional studies on the prevalence and incidence of anemia and iron-deficiency in different type of AF subgroups, including by age, gender, anticoagulation use, ethnicity, and clinical setting.
Clinical symptoms	Impact of anemia and iron deficiency on AF symptoms, functional capacity including exercise and peak oxygen consumption in patients with AF
Pathophysiology	Assessment of iron-deficiency as contributors to anemia in patients with AF
Treatments	Evaluation of treatment effects with iron supplements on anemia and iron-deficiency in patients with AF
Side effects	Confirmation of reported associations of anemia, stroke, and bleeding in patients with AF Impact of iron deficiency on future complications such as stroke, bleeding, heart failure, and mortality

## 8 Conclusion

Both anemia and ID are highly prevalent in individuals with AF. Moreover, anemia and ID may be associated with worsening symptoms and outcomes in patients with AF. Future studies will be required to confirm the prevalence of anemia and ID across different populations with AF, better characterize the associations with outcomes, and ultimately determine whether correction of anemia and ID is a novel management strategy for patients with AF.
