# Supplementary material for: Advances in iron deficiency and iron-related arrhythmias and cardiovascular diseases
Source: Front Cardiovasc Med. 2025 Jul 11;12:1573095. doi: 10.3389/fcvm.2025.1573095 (PMC12289698; doi:10.3389/fcvm.2025.1573095)
Supplement: Supplementary file 1 [file Image1.pdf]

Advances in Iron Deficiency and Iron-Related Arrhythmias and Cardiovascular diseases

Potential mechanisms linking atrial fibrillation, heart failure , iron deficiency, and anemia.

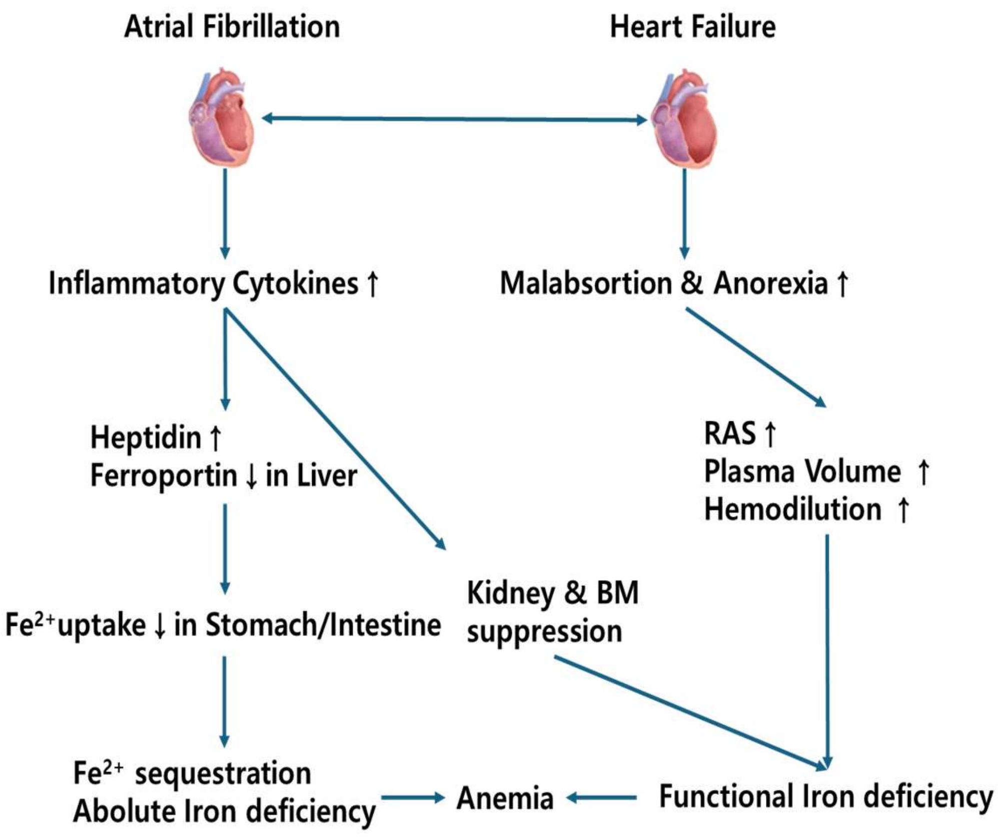

** RAS indicates renin angiotensin system; BM, bone marrow.

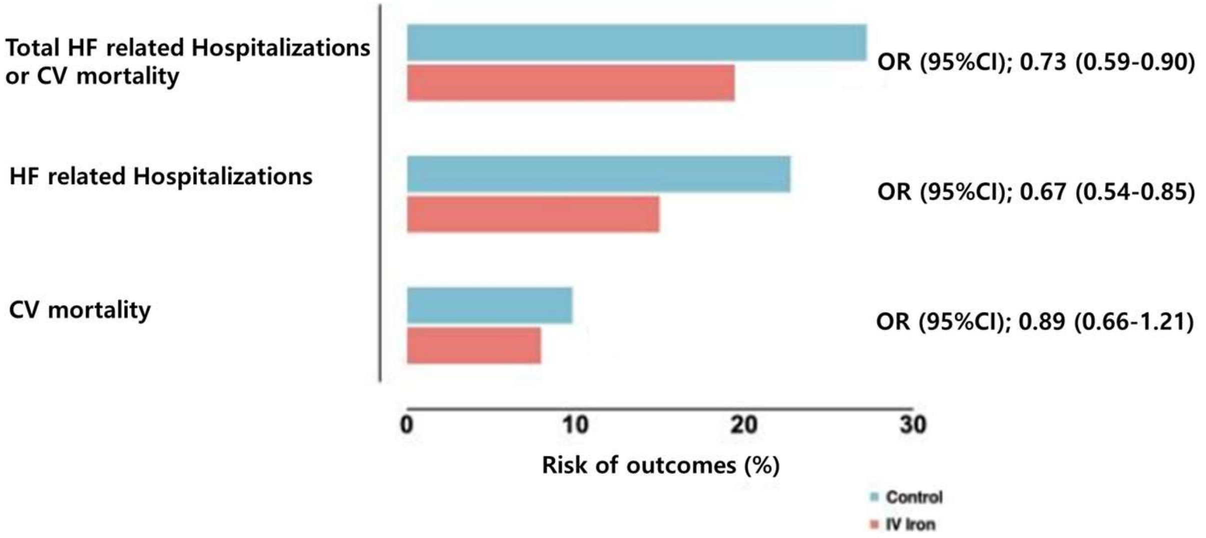

- Risk of cardiovascular outcomes with intravenous iron vs. placebo on intraven ous iron supplementation in HF with iron deficiency.
- Both anemia and ID are highly prevalent in individuals with AF. Moreover, anemia and ID may be associated with worsening symptoms and outcomes in patients with AF.

** HF indicates heart failure; CV, cardiovascular; OR, odds ratio; CI, confidence interval; ID, iron deficiency; AF, atrial fibrillation.
